# Protective efficacy of standard Edmonston-Zagreb measles vaccination in infants aged 4.5 months: interim analysis of a randomised clinical trial

**DOI:** 10.1136/bmj.a661

**Published:** 2008-07-31

**Authors:** Cesário L Martins, May-Lill Garly, Carlito Balé, Amabelia Rodrigues, Henrik Ravn, Hilton C Whittle, Ida M Lisse, Peter Aaby

**Affiliations:** 1 Bandim Health Project, Indepth Network, Bissau, Guinea-Bissau; 2 Bandim Health Project, Danish Epidemiology Science Centre, Statens Serum Institut, Artillerivej 5, 2300 Copenhagen S, Denmark; 3 MRC Laboratories, Fajara, Gambia; 4 Department of Pathology, Herlev University Hospital, Denmark

## Abstract

**Objective** To examine the protective efficacy of measles vaccination in infants in a low income country before 9 months of age.

**Design** Randomised clinical trial.

**Participants** 1333 infants aged 4.5 months: 441 in treatment group and 892 in control group.

**Setting** Urban area in Guinea-Bissau.

**Intervention** Measles vaccination using standard titre Edmonston-Zagreb vaccine at 4.5 months of age.

**Main outcome measures** Vaccine efficacy against measles infection, admission to hospital for measles, and measles mortality before standard vaccination at 9 months of age.

**Results** 28% of the children tested at 4.5 months of age had protective levels of maternal antibodies against measles at enrolment. After early vaccination against measles 92% had measles antibodies at 9 months of age. A measles outbreak offered a unique situation for testing the efficacy of early measles vaccination. During the outbreak, 96 children developed measles; 19% of unvaccinated children had measles before 9 months of age. The monthly incidence of measles among the 441 children enrolled in the treatment arm was 0.7% and among the 892 enrolled in the control arm was 3.1%. Early vaccination with the Edmonston-Zagreb measles vaccine prevented infection; vaccine efficacy for children with serologically confirmed measles and definite clinical measles was 94% (95% confidence interval 77% to 99%), for admissions to hospital for measles was 100% (46% to 100%), and for measles mortality was 100% (−42% to 100%). The number needed to treat to prevent one case of measles between ages 4.5 months and 9 months during the epidemic was 7.2 (6.8 to 9.2). The treatment group tended to have lower overall mortality (mortality rate ratio 0.18, 0.02 to 1.36) although this was not significant.

**Conclusions** In low income countries, maternal antibody levels against measles may be low and severe outbreaks of measles can occur in infants before the recommended age of vaccination at 9 months. Outbreaks of measles may be curtailed by measles vaccination using the Edmonston-Zagreb vaccine as early as 4.5 months of age.

**Trial registration** Clinical Trials NCT00168558 [ClinicalTrials.gov].

## Introduction

The World Health Organization’s policy for measles vaccination was established in the early 1980s after studies of antibody responses to vaccination at different ages.^1^ ^2^ The assumption was that children with antibodies were fully protected and those without measurable antibodies were fully susceptible and that vaccination at either 8 or 9 months of age would provide a similar proportion of protected children. It was thought, however, that vaccination at 8 months would lead to more children getting measles after vaccination, so called vaccine failures. Because it was believed that vaccine failures would lead to lack of confidence in the vaccination programme, vaccination at 9 months of age was finally recommended and became global policy. The policy makers did not take into consideration that by vaccinating earlier, measles infection would move to an older age group with a lower case fatality,^3^ that vaccinated children have partial immunity and milder disease,^4^ and that vaccine failures with milder disease do not discourage mothers from vaccination, rather the opposite.^4^ No trial was done to establish the evidence that by vaccinating at 6, 7, 8, or 9 months of age the effects on measles morbidity and overall mortality might differ.^3^

The current measles vaccination policy was based on children born to naturally infected mothers. Such children may not lose protection from maternal antibodies until around 7 to 9 months of age.^5^ ^6^ The situation has changed since the 1980s. Most low income countries have had measles vaccination programmes for the past 20-25 years. Guinea-Bissau carried out the first campaign in 1979.^7^ As a consequence many mothers have been immunised and have not had natural measles infection. In Guinea-Bissau and Senegal we examined the transfer of maternal antibodies from mothers with known measles vaccination or natural measles infection. In both sites immunised mothers transferred only around half the concentration of maternal antibodies, and their children lost protective levels early (unpublished data).^8^ ^9^ A new group of children born to immunised mothers therefore exists who may lose protection by 3 to 5 months of age. This situation should lead to reconsideration of the policy for measles vaccination.

In a search for a better policy we originally hoped that measles vaccination using high titre Edmonston-Zagreb vaccine at 6 months of age could solve the problem of measles control and improve the survival of children.^10^ ^11^ Because this vaccine was withdrawn ,^12^ we examined two dose strategies that provided the first dose before 9 months of age.^12^ ^13^ Two dose strategies increase the likelihood of getting at least one dose and thereby increase individual protection and herd immunity.^14^ Vaccination before 9 months of age should also protect young infants, who have a particularly high case fatality rate,^15^ and might extend the non-specific beneficial effects of measles vaccination by providing vaccine to a younger age group.^16^ ^17^ ^18^

Since the late 1990s spectacular successes in measles control have been obtained with supplemental immunisation activities, including mass campaigns targeting children aged 9 months to 14 years.^19^ ^20^ ^21^ After such campaigns in Latin America the age of routine vaccination has been increased to 12 months as vaccination at this age is believed to provide fuller protection^20^ and because the risk of exposure to measles virus is greatly reduced after supplemental immunisation activities. The success of the campaigns in Latin America^19^ ^20^ provides some support for this strategy. The optimal strategy in Africa may be different, however, because there are more births per woman, more urban crowding, a higher risk of early measles infection before the current age of vaccination, and less continuity in vaccination efforts. Even if current efforts for elimination are successful in Africa,^21^ it is likely that break-downs in vaccinations, political upheaval, and humanitarian crises^22^ will cause local outbreaks. In such situations vaccination against measles before 9 months of age may be desirable. Furthermore, delaying the age of vaccination would reduce the non-specific beneficial effects of the vaccine.^16^ ^17^

We have continued to examine possible modifications in the measles vaccination policy. In 2003 we started a trial of two doses of Edmonston-Zagreb measles vaccine at 4.5 and 9 months of age in urban Guinea-Bissau. We included only children who had received the third dose of diphtheria, tetanus, and pertussis vaccine before enrolment. Measles was present in Guinea-Bissau and shortly after the trial started an outbreak occurred in the study area and provided a unique situation for testing the efficacy of vaccination before 9 months of age.

## Methods

The trial was carried out in the study area of the Bandim Health Project, which covers five districts totalling about 90 000 inhabitants, 30% of the population of the capital Bissau.^23^ The largest of the areas, Bandim 1, has been followed since 1978; Bandim 2 and Belem were included in the surveillance system in 1983, Mindara in 1993, and Cuntum in 1998. The residents have an identification number in the census files, along with information on house number and socioeconomic and demographic factors. The houses are visited every month to register new pregnancies and births. All children are visited at home every three months until age 3 years; at these visits we collect information on breastfeeding status, infections, admissions to hospital, vaccination status, whether the mother lives with the child, and ownership of pigs, since these factors have been associated with survival or mortality in children. The project area has three health centres.

The women of Bissau travel often to visit relatives living in other parts of the country and to take part in the cashew harvest. About 20% of the mothers are travelling at any given time. A large proportion of the children therefore may come late for vaccinations. It is always possible to obtain information on survival, however, because if a child dies in the rural areas, the family in Bissau are informed immediately.

### Objectives, study design, and outcomes

The present trial was originally planned to compare different vaccination strategies using the two main strains of measles vaccine, Edmonston-Zagreb and Schwarz, which are used interchangeably in low income countries. The trial design included three arms: two dose measles vaccination with Edmonston-Zagreb vaccine at 4.5 and 9 months of age, Schwarz vaccine at 9 months of age, and Edmonston-Zagreb vaccine at 9 months of age. The primary end point was survival to 3 years of age. The children were enrolled at 4.5 months of age and randomised to one of the three groups. At 9 months of age the children were invited back to the health centre; the treatment arm received a second standard dose of Edmonston-Zagreb vaccine and the control arms received a standard dose of either Schwarz vaccine or Edmonston-Zagreb vaccine.

Several studies have suggested that diphtheria, tetanus, and pertussis given simultaneously with measles vaccine or after measles vaccination may be associated with increased mortality compared with the measles vaccine given alone.^18^ ^24^ ^25^ ^26^ For example, in the high titre measles vaccination trials the excess mortality in girls may have been due to the diphtheria, tetanus, and pertussis vaccine being given after the measles vaccine.^9^ We made it a condition that children in the present trial should have received three doses of the diphtheria, tetanus, and pertussis vaccine before enrolment. Allowing for an interval of four weeks between the three dose schedule and measles vaccination, 4.5 months of age was the earliest we could give the measles vaccine.

The primary outcomes were survival at 3 years of age, levels of measles antibodies at 24 months of age to assess likely protection against measles infection, and protection against measles infection. The trial was started in August 2003 and follow-up is still ongoing. We expect to report later the data on measles antibody levels and survival. We are currently in add-on studies assessing the cytokine response after early measles vaccination and the response to a booster vaccination at 3 years of age to predict the likely long term efficacy of early measles vaccination.

### Sample size

The sample size was based on the calculation that to detect a 25% difference in mortality between any of the groups we needed to enrol 5755 children and to follow them to 3 years of age. For the evaluation of antibody levels we calculated that we needed 400 children to detect a 10% difference in the proportion of those showing no seroconversion. No sample size was calculated for protection against measles infection. We have maintained measles surveillance for the past 25 years in part of the study area and we did not expect sufficient numbers of measles cases to assess the impact of the various strategies on the incidence of natural measles infection.

### Interim analysis

Contrary to expectations a large outbreak of measles occurred between November 2003 and May 2004, shortly after we started the trial. Given the noticeable efficacy of early measles vaccination one of the participating ethical committees questioned the ethics of continuing the trial. The investigators decided after consultation with the trial data safety monitor (Robin Bailey) and the ethics committees that the study could continue because of the limited possibilities for further outbreaks of measles. Should a new outbreak occur after the interim analysis, the main study would be stopped temporarily and all unvaccinated children be offered the Edmonston-Zagreb measles vaccine at 4.5 months of age.

It was also decided to report immediately the efficacy of early measles vaccination. In contrast to the three groups specified in the protocol, this interim analysis would directly compare two groups: children receiving the Edmonston-Zagreb vaccine at 4.5 months of age and the combined group of children to receive the Schwarz vaccine or Edmonston-Zagreb vaccine at 9 months of age. Whereas overall mortality until 3 years of age was the primary outcome in the planned trial, the incidence of measles, admissions to hospital for measles, and measles mortality were the primary outcomes in the interim analysis. Hence the interim study reports the efficacy of early vaccination with the Edmonston-Zagreb vaccine in an outbreak situation. The study includes all children enrolled in the trial between the start of the study and the end of May 2004, when the measles epidemic ended. The present analysis (fig 1) deals with the effect of Edmonston-Zagreb measles vaccination only before the 9 month vaccination; follow-up was censored if the children received measles vaccine, moved, died, or were at the end of the epidemic (1 June 2004). The original sample size calculation was not relevant to the interim analysis but the study procedures were the same in the subgroup included in the interim analysis.

**Figure fig1:**
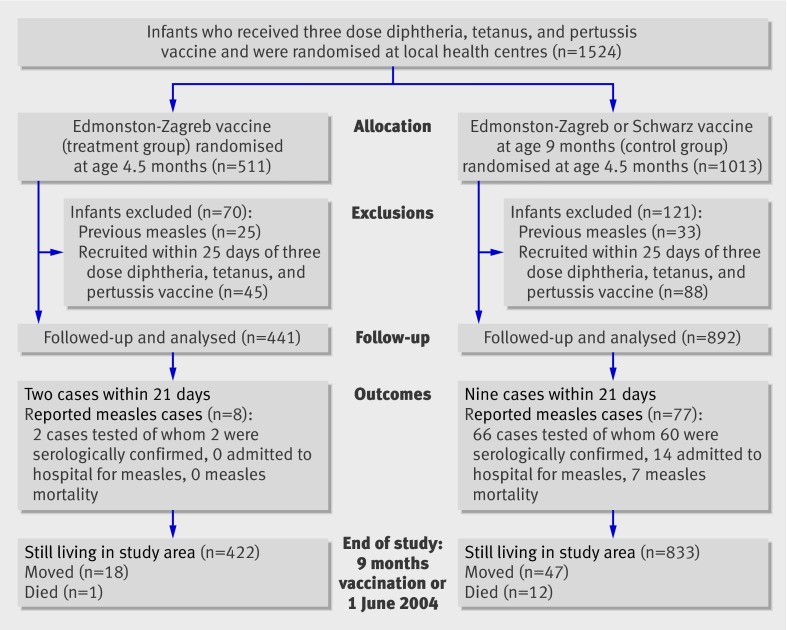
**Fig 1** Flow of children through trial. Numbers of children with measles are not mutually exclusive categories but represent different degrees of diagnostic certainty (serologically confirmed) or clinical severity (admission to hospital, death)

### Enrolment

Newborn infants were identified through the Bandim Health Project’s registration system. To make sure that study children had received the third dose of diphtheria, tetanus, and pertussis four weeks before inclusion we contacted the mothers of children aged 6, 10, and 14 weeks and reminded them to go to one of the health centres to have their children vaccinated and given the oral polio vaccine. Children were enrolled at 4.5 months old. In the morning a fieldworker contacted the mothers or guardians of eligible children, explained the study, filled in a questionnaire on background factors, and obtained verbal consent. Mothers were asked to bring their children to the health centre in the afternoon.

In the afternoon a doctor provided the mothers or guardians of children presenting at the health centre with an oral and a written explanation of the study. At each visit to the health centre the children were examined by the doctor. Clinical examination and treatment was independent of randomisation group. The children received treatment according to local standards. Children who were sick and needed to be admitted to hospital could only participate in the study when they had recovered.

### Randomisation and intervention

The data manager, who was not involved in the recruitment of children, prepared bags with 24 numbered envelopes indicating the randomisation group. These numbers could not be seen by the doctor informing and obtaining consent from the mothers or guardians.

If consent to participate was given, the mothers or guardians were asked to select one of the envelopes. This procedure has been used in several other trials in the study area and emphasises to mothers that they are participating in a randomised trial. The children were randomised to one of three equally sized groups, using block randomisation with 24 envelopes per bag. A new bag was opened only when the previous one was empty.

Children randomised to Zagreb-Edmonston vaccination at 4.5 months of age received a standard titre from the Serum Institute of India, Pune, India (batch number 2360).

### Masking

No placebo was given. Ethical committees in Africa have been reluctant to use a placebo injection in vaccine trials,^27^ usually preferring some potentially beneficial vaccination (for example, rabies or meningococcal vaccine), but irrelevant to the main outcome of the trial. We have used inactivated polio vaccine as a control vaccine in previous trials of early measles vaccination.^18^ As reported recently, however, such a vaccine may have an impact on the female to male mortality ratio.^18^ It was therefore preferable not to have another active control vaccine. Furthermore, if we had used a placebo some mothers in the control group might erroneously have believed that the child had received a measles vaccine despite it not being noted on the vaccination card. As long as participants remained in the study area this mattered little as they would all be called systematically for measles vaccination at 9 months of age. If they moved from the area, however, they might not have gone to a health centre to seek measles vaccination. Mothers of children in the control group were told that their children would not receive measles vaccine at enrolment but that they should be vaccinated at 9 months of age.

### Outcomes

#### Maternal antibody levels and seroconversion after early vaccination

We collected blood samples to assess the levels of maternal antibody levels against the measles virus and antibody levels after different measles vaccination strategies. We collected samples at 4.5, 9, and 24 months of age from the early vaccination group and at 9, 18, and 24 months of age from the control groups. Some of these samples were collected from children during the measles epidemic (see table on bmj.com). These samples have been used to indicate the level of maternal antibodies at 4.5 and 9 months of age within this cohort. Furthermore, the samples at 4.5 and 9 months of age in the treatment group have been used to document the degree of seroconversion after early measles vaccination. We excluded children who had measles before sampling or who had known exposure to measles at home.

#### Measles surveillance

A measles outbreak built up during the autumn of 2003 and lasted until May 2004. During that time the Bandim Health Project offered free consultations and treatment for children with measles, both at the project’s premises and at the paediatric ward in the national hospital, Bissau. Children with measles seen at the local health centres in the study area were identified. Those children identified were visited at home to offer treatment and to identify new cases. The houses were visited by fieldworkers as part of their routine activities to identify new pregnancies and to follow children under 3 years. Fieldworkers attempted to identify additional cases of measles at these visits.

Several special activities for data collection were started during the outbreak. Children under 5 years were visited in March or April 2004 after the peak of the outbreak to obtain information on measles cases. Two special surveys to obtain information on measles infection or exposure to measles infection were carried out for children enrolled in the present trial, in the early phase in January 2004 and at the end of the outbreak, in June or July 2004. These surveys were carried out by fieldworkers who were not involved in the enrolment of children. At enrolment and at vaccination at nine months mothers or guardians were asked whether the children had had measles. Children not previously identified with measles were visited at home by a doctor to collect information on symptoms and exposure.

#### Measles diagnosis

We used both clinical and serological criteria to define the diagnostic quality of children reported with measles during the outbreak. Children examined by the project doctors or at the paediatric ward were considered to have definite clinical measles if they had Koplik’s spots or a typical measles rash, or both, or a history of measles with typical desquamation simultaneous with less specific symptoms of infection, including fever, cough, respiratory infection, stomatitis, or conjunctivitis. Children reported as having measles but who were not seen in the acute phase were visited at home by a doctor and were considered to have probable measles if the family reported symptoms of rash, desquamation, cough, and conjunctivitis, and contacts with known children with measles infection. We included both definite and probable cases of measles in the analysis of measles incidence.

We collected blood samples from children in the acute phase of measles and one month later for determination of antibody levels. For children seen only in the second month after measles infection a blood sample was collected for possible confirmation with an IgM antibody test.

More than 1750 cases of measles were reported in the study area in 2003-4. During the most intensive months of the outbreak, from December 2003 to March 2004, the three study doctors were overwhelmed by the management of cases at the project and health centres. For this reason many cases not seeking care were not seen in the acute phase but identified later through our surveillance systems. Children brought to the project or health centres were therefore more likely to have a diagnosis of definite clinical measles than children identified through the community’s registration system.

#### Admission to hospital for measles

The Bandim Health Project registers children admitted to the national hospital in Bissau, which has the only paediatric ward in the country. Overall, 905 children with measles were admitted to hospital from the city of Bissau during the outbreak. From the vaccination card we obtained sufficient information to identify the children in the project’s main registration system.^28^ ^29^

#### Measles mortality

Children with measles were followed to ascertain deaths in the acute phase—defined as within one month (31 days) of the onset of rash.

#### Concealment of vaccination status and identification of measles cases

The fieldworkers carrying out the surveys on measles infection or exposure to measles in the study population were unaware of the vaccination status of the children and had not taken part in enrolment. The doctor carrying out the clinical examination of suspected cases was asked to avoid looking at the vaccination card before making a diagnosis. At the paediatric ward, the doctors admitting the children would not look at the vaccination card and ascertainment may therefore have been relatively blind at the hospital.

#### Testing for measles antibodies

The Medical Research Council Laboratories in the Gambia carried out IgM and IgG testing for measles antibodies as previously described.^30^ The sensitivity of the IgG measles haemagglutination inhibition assay is 15.6 mIU, the minimum detectable level is 31.2 mIU, and the protective level is considered to be 125 mIU or greater.^31^ A fourfold increase in IgG titre or a positive IgM test result was interpreted as confirmation of acute measles infection. Because of the decline in the IgM response in the second month after measles infection, we did not interpret negative IgM responses in convalescent sera as invalidating a measles diagnosis. Only negative IgG responses in convalescent sera were considered to invalidate a measles diagnosis.

#### Statistical analyses and vaccine efficacy

Many children had had measles before enrolment. As they were not susceptible to the intervention we excluded such children from the present analysis (fig 1). Children with measles soon after enrolment were likely to have contracted infection before enrolment, and vaccination would not necessarily have prevented infection in the context of intensive exposure to measles virus. Thus we excluded from the main analysis children with measles occurring within 21 days of enrolment. Likewise, we followed up children to 21 days after the measles vaccination at 9 months of age as this vaccination is unlikely to have prevented infection within that period. We calculated vaccine efficacy against measles infection, admissions to hospital for measles, and measles mortality from the incidence rate ratio between treated and control children using the formula: vaccine efficacy=(1−incidence rate ratio)×100.^32^ We estimated the incidence rate ratio using a Cox proportional hazards model, with time since enrolment as the time variable.

The protocol specified that children should be enrolled four weeks after the three dose vaccination schedule against diphtheria, tetanus, and pertussis. In the first month, however, a programming error meant that 133 children were enrolled shortly after receiving the three dose schedule. In the main analysis we censored children who were enrolled within 25 days of the three dose vaccination. Results are also presented with inclusion of these children.

## Results

Between August 2003 and May 2004 1524 children were enrolled in the study; 133 were recruited within 25 days of the three dose diphtheria, tetanus, and pertussis vaccination schedule and were therefore not included in the main analysis (fig 1). Among the remaining 1391 children, 58 had been seen with measles or reported measles before enrolment and were therefore no longer at risk of infection. Overall, 1333 children were included in the analysis of the protective effect of early measles vaccination using the Edmonston-Zagreb vaccine; 441 in the group treated at 4.5 months (treatment group) and 892 in the two control groups due to receive the first dose of measles vaccine at 9 months of age.

### Baseline data

Children in the treatment and control arms were similar for demographic, socioeconomic, and health related factors (table 1). The median delay between the three dose diphtheria, tetanus, and pertussis vaccination and enrolment was 32 days (25-75% centiles 29-35). The median age at enrolment was 4.8 months (25-75% centiles 4.7-5.1); 871 (65.3%) were aged 4 months, 427 (32.0%) 5 months, and 35 (2.6%) 6 months. Of the 188 children in the treatment arm tested for maternal antibodies at enrolment (see table on bmj.com), 61% (n=114) had detectable levels but only 28% (n=52) had protective levels, the prevalence being lower among girls than among boys (20% *v* 35%; relative risk 0.55, 95% confidence interval 0.34 to 0.91). At 9 months of age, only 5% (21/405) of the control children had protective antibody levels.

**Table 1 tbl1:** Characteristics of children allocated to Edmonston-Zagreb measles vaccine at age 4.5 months (treatment) or to Schwarz or Edmonston-Zagreb vaccine at age 9 months (controls). Values are percentages (numbers) unless stated otherwise

Characteristic	Treatment arm	Control arm
No of children	441	892
Median age (months) at enrolment (25-75% centiles)	4.8 (4.7-5.2)	4.8 (4.7-5.1)
Median age (years) of mother (25-75% centiles)	25 (21-29)	25 (20-29)
Resident of Bandim district	41 (182)	45 (398)
Boys	48 (211)	50 (442)
Twins	3 (15)	3 (22)
Pepel ethnic group	30 (111)	28 (210)
Risk factors at enrolment (age 4.5 months):		
Not being breast fed	2 (8)	3 (25)
Pigs in household	16 (71)	13 (119)
Pigs in house	32 (141)	29 (261)
Average No of people per bed	3.0	3.0
No of people per bedroom	4.2	4.3
Inside toilet	16 (72)	14 (127)
Electricity	42 (184)	42 (378)
Functioning electricity	26 (113)	25 (222)
Morbidity and anthropometry:		
Admission to hospital before age 4.5 months	1 (5)	1(5)
Fever	10 (46)	11 (101)
Diarrhoea	5 (22)	5 (46)
Mean respiratory rate	42	43
BCG scar	82 (362)	82 (728)
Chloroquine at home	29 (129)	27 (236)
Mean (SD) weight (g)	7109 (956)	7140 (979)
Mean (SD) arm circumference (mm)	140 (11)	141 (12)
Mean (SD) height (cm)	64.1 (2.9)	64.4 (2.8)
Mean (SD) mother’s arm circumference (mm)	270 (34)	270 (33)

### Outcomes

#### Antibody levels after early vaccination

Antibody levels were determined in 544 children aged 9 months who had not had measles infection and had no known exposure to measles infection at home (see table on bmj.com). Among children in the treatment group, 92% (128/139) had a measurable haemagglutination inhibition antibody titre compared with 9% (38/405) among the control children (relative risk 9.81, 95% confidence interval 7.2 to 13.3); 77% (107/139) and 5% (21/405), respectively, had protective levels (14.9, 9.7 to 22.7).

#### Diagnosis of measles infection

Overall, 103 children were reported as having measles between enrolment and within three weeks of measles vaccination at 9 months of age. Seven children had one or two negative convalescent samples and were invalidated. Therefore 96 children had a diagnosis of measles on the basis of clinical or serological data; 48 (50%) were seen in the acute phase and considered to have definite clinical measles and the remaining 48 were classified as having probable measles. Sixty nine children (72%) were serologically confirmed as having definite measles, with a fourfold increase in antibody levels or a positive response with the IgM test. Of the remaining 27 children, eight unvaccinated children had high IgG antibody levels but were sampled too late to be IgM positive and no adequate sample was collected from 19 children, mainly because the child had died, was admitted to hospital, had travelled, or was seen only after the measles vaccination at age 9 months when it would be impossible to assess whether antibodies were due to measles infection or to vaccination. Eight of the children with no adequate blood sample had been considered to have clinically definite measles. Hence, 77 children (80%) had a serologically confirmed diagnosis or a clinically definite diagnosis.

#### Incidence of measles infection

The incidence of measles was high; 4.2% (58/1391) of the children had measles before enrolment at 4.5 months; 33 had serologically confirmed measles. These children were excluded from further analysis (fig 1). Among the children in the control group 86 contracted measles (fig 2). The monthly incidence was 3.1% (95% confidence interval 2.5% to 3.8%) and for children remaining unvaccinated the cumulative incidence reached 14.4% (11.7% to 17.7%) before 9 months of age and 19.4% (14.1% to 26.4%) before 12 months of age. Ten children in the treatment arm contracted measles (fig 2). The monthly incidence was 0.7% (0.4% to 1.3%) and the cumulative incidence was 3.1% (1.5% to 6.2%) before 9 months of age and 6.7% (2.6% to 16.4%) before 12 months of age. The case fatality for all cases was 8.3% (8/96).

**Figure fig2:**
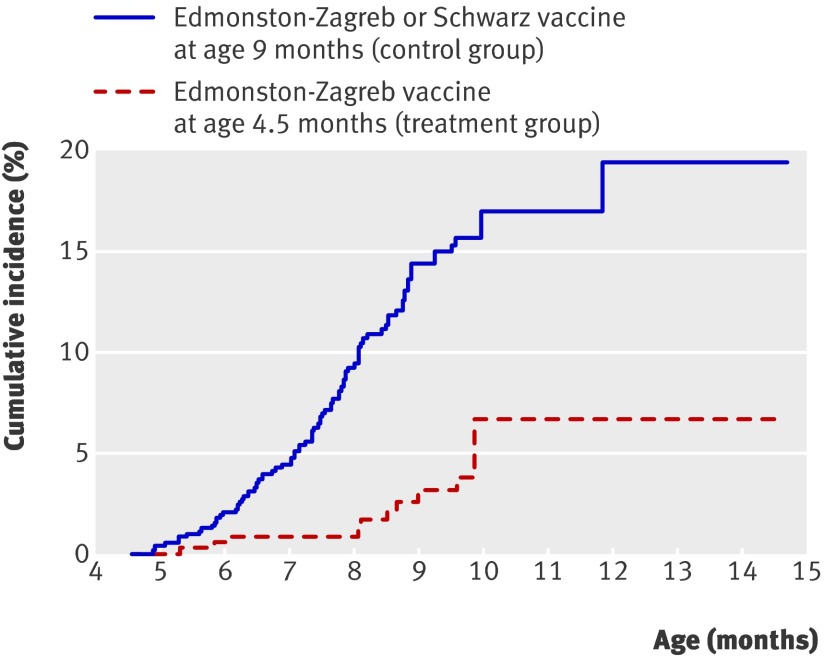
**Fig 2** Cumulative incidence of measles in children receiving early immunisation against measles using Edmonston-Zagreb vaccine at age 4.5 months (treatment) and Schwarz vaccine or Edmonston-Zagreb vaccine at age 9 months (controls). Of 86 children who contracted measles in the control group two were among infants aged 4 months, 12 aged 5 months, 17 aged 6 months, 28 aged 7 months, 21 aged 8 months, five aged 9 months, and one aged 11 months. Of 10 children with measles in the treatment group two cases occurred among infants aged 5 months, one aged 6 months, five aged 8 months, and two aged 9 months.

#### Vaccine efficacy

Eleven children had measles within 21 days of enrolment and as they might have been infected before enrolment these children were excluded from the analysis (fig 1). Table 2 shows vaccine efficacy for the remaining 85 children for those with serologically confirmed measles, those admitted to hospital for measles, and for measles mortality. The vaccine efficacy for serologically confirmed cases was 94% (74% to 98%) and for clinically definite cases was 91% (62% to 98%). When serologically confirmed and clinically definite cases were combined vaccine efficacy was 94% (77% to 99%). Efficacy was as good for those enrolled at 4 months of age as for those enrolled at 5 or 6 months of age (data not shown). The number needed to treat to prevent one child having measles between 4.5 and 9 months of age in an epidemic situation was 7.2 (95% confidence interval 6.8 to 9.2).

**Table 2 tbl2:** Vaccine efficacy against measles, admission to hospital for measles, and measles mortality between three weeks after enrolment to study and three weeks after measles vaccination at age 9 months, Guinea-Bissau 2003-4

Events	Treatment arm*	Control arm†	Vaccine efficacy (%)
Diagnostic certainty:			
All measles cases	8	77	80 (59 to 90)
Serologically confirmed cases	2	60	94 (74 to 98)
Clinically definite cases	2	41	91 (62 to 98)
Serologically confirmed or clinically definite cases	2	66	94 (77 to 99)
Severe measles:			
Admission to hospital	0	14	100 (46 to 100)
Measles mortality	0	7	100 (−42 to 100)
Case fatality (deaths/cases)	0/8	7/77	

Five of these cases occurred after 9 month vaccination but within 21 days of vaccination: two in Edmonston-Zagreb vaccine group (4.5 months), and three in control group—one in Schwarz group and two in Edmonston-Zagreb group (9 months).

*Edmonston-Zagreb measles vaccination at age 4.5 months.

†Measles vaccination using Schwarz vaccine or Edmonston-Zagreb vaccine at age 9 months.

All admissions to hospital for measles occurred among children in the control arm; vaccine efficacy against admission was 100% (46% to 100%; table 2). Apart from admissions for measles, 21 children in the control arm and eight in the treatment arm were admitted. Therefore the treatment group had no more admissions to hospital for non-measles related reasons than the control group.

The seven deaths from measles that occurred more than 21 days after enrolment were in the control arm; vaccine efficacy against death was 100% (−42% to 100%). From the start of the trial to the end of the measles outbreak, overall mortality was lower in the treatment arm (fig 1) although the mortality rate ratio of 0.18 (0.02 to 1.36) was not statistically significant. Apart from the seven deaths from measles (table 2, five more children in the control groups died with a diagnosis of measles (had measles within the first 21 days), sepsis, or respiratory tract infection, and two died from severe malaria. One child in the treatment arm died as a result of diarrhoea and vomiting. Therefore the treatment group had no more non-measles related deaths than the control group.

These estimates were not materially changed by inclusion of children who contracted measles in the 21 days after enrolment or among children who had been enrolled too soon after the three dose vaccination schedule against diphtheria, tetanus, and pertussis. The vaccine efficacy for confirmed cases was 92% (74% to 97%), against admission to hospital was 100% (45% to 100%), and against death was 100% (−7% to 100%; P=0.034).

## Discussion

Standard titre Edmonston-Zagreb measles vaccination at 4.5 months of age in Guinea-Bissau was protective against measles infection and admission to hospital for measles. For children with definite measles, protection was more than 90%. Most of the cases were serologically verified and, even if we included all self reported cases, vaccine efficacy was still 80%. The study was too small to document significant protection against measles mortality. Such a protection is, however, likely as admission to hospital is a reliable indicator of the severity of infection and in the analysis that included children with measles occurring within 21 days of enrolment and children who had been enrolled too soon after the three dose diphtheria, tetanus, and pertussis schedule, the difference in measles mortality was also significant. Furthermore, overall mortality may have been somewhat lower in the treatment group than control group although this was not statistically significant.

### Strengths and weaknesses

The present analysis is an interim report from a large randomised trial so conclusions should be interpreted with caution. It is unlikely that further cases of measles that may modify the estimate of vaccine efficacy will occur before the end of the trial. The epidemic raised herd immunity, and a national measles immunisation campaign of children aged 6 months to 15 years was carried out in May 2006. The efficacy of early Edmonston-Zagreb measles vaccination presumably depends on the level of maternal antibodies and that could differ in other populations in low income countries.^33^

The most important limitations of the study may relate to the lack of blinding of vaccination status among possible measles cases and to the impossibility of seeing all children in the acute phase of infection. Firstly, surveys to identify cases in the community and the detection of cases at the health centres and the hospital were carried out without reference to the vaccination status. Although the project doctors were under instruction not to look at the vaccination card before diagnosis we cannot be certain they never looked. Nearly all diagnoses were confirmed in the independent blinded serological analyses. The selection of children from whom blood samples were collected could have been biased. We think this is unlikely as Guinean doctors have often observed measles infection in vaccinated children and are therefore unlikely to be influenced by assumptions about children vaccinated against measles not getting the infection. At the paediatric ward, the doctors who admitted the children would not inspect the vaccination card and ascertainment at the hospital has been relatively blind. The hospital data clearly showed good protection from Edmonston-Zagreb measles vaccination at 4.5 months of age. Had an important bias affected the decision to admit a child with possible measles to hospital, we should have seen in the treated group relatively more admissions to hospital for non-measles related reasons and for deaths not related to measles. That did not occur. If anything fewer children in the treated group were admitted to hospital for “non-measles” and died. It would seem unlikely that possible bias in the diagnostic process has had a major impact on our identification of children with measles.

Secondly, not all children with measles could be seen, partly because the population is mobile and infected children may have been absent. More importantly, the workload during the epidemic made it impossible for clinicians to search for children with mild infection who did not turn up for consultation. This could have meant that we identified too few cases, particularly children with milder disease. During the epidemic fieldworkers who were unaware of the randomisation status of the children carried out several surveys to identify children with measles. These surveys identified most of the children with probable measles. This process provided several opportunities for mothers to report that their child had had measles. We therefore think it unlikely that many cases remained unreported and undetected. The incidence of measles was high, with 4.2% of the children having measles before enrolment and an additional 14.4% before 9 months of age in the control arm.

### Critical features of the intervention

We were surprised that vaccine efficacy was so high. Maternal antibody levels would presumably interfere with vaccination at 4.5 months of age. Only 28% of the children had protective antibody levels at enrolment, however, and the Edmonston-Zagreb vaccine may be able to immunise in the presence of maternal antibodies.^10^ ^13^ ^30^ Nearly all of the children receiving the Edmonston-Zagreb vaccine had measurable antibodies at 9 months of age and would be protected or have had only mild infection.^31^ These results apply only to Edmonston-Zagreb measles vaccine, which may immunise better in the presence of maternal antibodies than the Schwarz strain of measles vaccine, or the Moraten strain, which has been used with limited success in 6 month old American infants.^34^

### Changes in the epidemiology of measles infection

The major outbreak of measles occurred in an area that has had intense vaccination activities for many years. Mobility is high in this urban area and even though we have maintained over 90% coverage^13^ many children move away and other unvaccinated older children may have moved in. We did try to prevent an outbreak by recommending more general vaccination in connection with a vitamin A and missing vaccination campaign carried out by local health authorities in November 2003. The international organisations emphasised that international policy for measles vaccination was vaccination before 12 months of age and that infants only less than 12 months of age were to receive measles vaccine.

The epidemiology of measles may have changed fundamentally in recent years. To our knowledge no study has documented an incidence of 18.6% before 9 months of age in an area with high vaccination coverage. Measles is still a severe disease. The case fatality was high despite intensive surveillance, home visits, and follow-up by doctors, and free treatment at the hospital. Many low income countries have had a high coverage for measles vaccination for the past 20 years, since the universal childhood immunisation activities in the mid-1980s, which reached even the remotest part of Africa.^35^ Increasing numbers of mothers have been immunised in childhood, and re-exposure to the virus may have been limited. Therefore newborn infants will have lower antibody levels and are likely to become susceptible to measles infection and responsive to measles vaccination much earlier.^33^ This situation is further aggravated by the HIV epidemic because mothers infected with HIV transmit lower levels of measles antibodies to their offspring than uninfected mothers. The low maternal antibody levels detected in this study are likely to be representative of many low income countries. Severe outbreaks may occur in cities in which surveillance is less intense and access to care more difficult than in the Bissau setting.

### Policy implications

WHO currently recommends early two dose measles vaccination at 6 and 9 months of age during measles outbreaks, in humanitarian emergencies, and in refugee camps. Immunisation campaigns in response to outbreaks have often targeted children aged 6 months to 15 years.^36^ ^37^ Given the strongly beneficial effect of the vaccination strategy we tested, it should be possible to recommend the first dose of Edmonston-Zagreb measles vaccine at 4.5 months of age in situations with a high risk of infection before 9 months of age. In situations where control of outbreaks is needed, the Edmonston-Zagreb vaccine could be used to immunise young household contacts who may have some maternal antibodies but not enough to prevent them from becoming infected when exposed at home^15^ and to transmit the virus. If elimination of measles is planned it will be necessary in Africa to immunise as early as possible for many years. Several groups are working to produce new measles vaccines that may provide such early protection—for example, using BCG or fowlpox as vaccine carriers. The Edmonston-Zagreb vaccine may also be useful in this context. Any potential new measles vaccine used before 6 months of age should be tested against the Edmonston-Zagreb vaccine, the only vaccine with proved efficacy in this age range.

In conclusion, we suggest that an early two dose strategy providing Edmonston-Zagreb vaccination as early as 4.5 months of age might be used in humanitarian emergencies or during outbreaks with a high risk of measles infection. Whether such vaccination at 4.5 months can be recommended more generally as the primary measles vaccination in a two dose strategy might depend on the long term maintenance of protective levels. We will continue to follow the cohort in Bissau to assess long term maintenance of antibody levels. It would be convenient if the first of the two dose strategy could be combined with the third diphtheria, tetanus, and pertussis vaccination. Several observational studies suggest, however, that the combination of measles vaccine and diphtheria, tetanus, and pertussis vaccine has a negative effect on overall survival.^24^ ^25^ ^26^ Whether these vaccines can be combined requires testing in a randomised trial. The strategy with early measles vaccination should be studied in other sites as well.

Given international financial support, supplemental immunisation activities are likely to continue in even the poorest African countries. It might be thought that measles can be controlled in Africa in the same way it has been controlled in Latin America and that an early two dose strategy is not necessary. Supplemental immunisation activities are, however, unlikely to control measles fully,^21^ and local outbreaks will continue to occur. With the changing antibody profile of mothers in Africa, the high birth rate, and rapid urbanisation, African cities will be open to major outbreaks of measles should there be political upheaval or financial problems leading to failure to maintain a high measles vaccination coverage. An early two dose measles vaccination strategy, if not associated with a subsequent loss of protection at older ages, could strengthen the current efforts to control measles and reduce measles mortality. Hence a reduction in the age of routine measles immunisation and possibly a two dose strategy should be considered.

**What is already known on this topic**

Mothers immunised against measles transmit low levels of measles antibodies to their offspring
Measles vaccination using standard titre Edmonston-Zagreb vaccine might immunise children before 9 months of age even in the presence of maternal antibodies

**What this study adds**

In this interim analysis standard titre Edmonston-Zagreb measles vaccination at 4.5 months of age provided more than 90% protection against infection and 100% protection against admission to hospital

## Notes

We thank Mario Monteiro (Laboratório Nacional de Saúde Pública, Guinea-Bissau) and Elishia Roberts and Tibeh Faye-Joof (Medical Research Council Laboratories, the Gambia) for collection and analyses of blood samples.

Ethical approval: The protocol was approved by the Danish central ethical committee, the Gambia/Medical Research Council scientific and ethics committees, and the Guinean Ministry of Health’s research coordination committee. Participants had access to free consultations at the local health centres and to essential drugs free of charge.

Provenance and peer review: Not commissioned; externally peer reviewed.

## Notes

**Cite this as:** *BMJ* 2008;337:a661
